# Differential age-related patterns of Human Papillomavirus infection among women in Western Province, Zambia

**DOI:** 10.1371/journal.pgph.0006955

**Published:** 2026-11-12

**Authors:** Masiliso A. Liamba, David Chisompola, Charity Mukutuma, Benson M. Hamooya, Grace Kahenya, Sepiso K. Masenga

**Affiliations:** 1 School of Medicine and Health Sciences, University of Lusaka, Lusaka, Zambia; 2 Department of Cardiovascular Science and Metabolic Diseases, Livingstone Center for Prevention and Translational Science, Livingstone, Zambia; 3 HAND Research Group, School of Medicine and Health Sciences, Mulungushi University, Livingstone, Zambia; 4 Public Health Unit, Copperbelt University, Kitwe, Zambia; University of Michigan, UNITED STATES OF AMERICA

## Abstract

Human papillomavirus (HPV) infection remains a major global public health concern and is the leading cause of cervical cancer, particularly in low- and middle-income countries. Zambia has one of the highest cervical cancer burdens worldwide, yet evidence on age-specific determinants of HPV infection remains limited. This study assessed the prevalence and determinants of HPV infection among women aged ≤40 years and ≥41 years in Western Province, Zambia. A cross-sectional study was conducted between October 2025 and January 2026 among women attending cervical cancer screening clinics in Western Province. A total of 1,132 participants were recruited using consecutive sampling. Sociodemographic, behavioural, and reproductive data were collected using structured questionnaires. Exfoliated cervical samples were collected and tested for high-risk HPV genotypes using the Hologic Panther platform with transcription-mediated amplification (TMA). Mixed-effects logistic regression was used to identify factors associated with HPV infection while accounting for clustering at the health facility level. Overall, HPV prevalence was 33.6%. HPV prevalence was comparable between women aged ≤40 years and those aged ≥41 years (33.6% vs. 33.4%). Among women aged ≤40 years, multiple lifetime sexual partners, high parity, lack of HPV vaccination, contraceptive use, low income, single marital status, and rural facility location were independently associated with HPV infection (P < 0.05). Among women aged ≥41 years, high parity, low HPV knowledge, unemployment, low income, and geographic location were independently associated with HPV infection (P < 0.05). Spatial variation was also observed, with significantly higher infection rates in Mwandi district. HPV infection remains highly prevalent across, in both younger and older women. Although the determinants differ by age group, the substantial HIV burden and ongoing HIV transmission may contribute to the persistence of HPV infection. These findings support age-specific prevention strategies that integrate HPV vaccination, cervical cancer screening, health education and treatment services to reduce the burden of HPV infection.

## Introduction

Despite several interventions to combat human papillomavirus infection, the prevalence remains unacceptably high [1]. Human papillomavirus infection remains a major public health concern worldwide [2]. HPV is transmitted and is linked to cervical cancer in women, particularly in low and middle-income countries [3]. The natural course of HPV infection is typically transient and asymptomatic in the majority of individuals, and the infection clears with the immune system’s help, without clinical intervention, within 1–2 years [4]. Persistent infection with high-risk genotypes can result in the development of cervical intraepithelial and invasive cervical cancer. Approximately 604,127 new cases of cervical cancer and 341,831 deaths occurred globally in 2020 and 90% of deaths were from Sub-Saharan Africa, resulting in an age-standardized incidence of 13.3 per 100,000 women per year, while the mortality rate was 7.2 per 100,000 women per year [5].

Recent studies conducted in Zambia indicate variation in HPV prevalence by province, with rates estimated at 36% in Southern Province and as high as 65% in Lusaka Province [6,7]. This demonstrates a high burden of infection contributing to elevated rates of cervical cancer and associated mortalities. Globally, Zambia is ranked as the third highest burden of cervical cancer, with an age-standardized incidence rate of 65.5 to over 70 cases per 100,000 women and a mortality rate of 43.4 per 100,000 women in 2020 [8]. By 2023, approximately 3,000 new cervical cancer cases annually, with over 1,900 cases resulting in death, were recorded in Zambia, underscoring the severe and persistent public health challenge posed by cervical cancer in the country [9].

HPV infection alone cannot lead to the development of cervical cancer without the combination of other factors, such as age, parity, sexual debut, and long-term hormonal exposure [10]. These factors may contribute to persistent viral infection. Long-term hormonal exposure and high parity have been associated with cervical epithelial changes and immune modulation, creating a suitable environment that may support persistent HPV infection.

Studies indicate high HPV prevalence in women under 35, with a U-shaped trend reappearing around age 50 [11].The high prevalence of HPV infection may be associated with different factors, such as high sexual activity, knowledge gaps, low-income status and low vaccination rates. A study conducted in China showed that the age prevalence of HPV was highest in younger women [12] and in South Africa, the study reported the highest prevalence among women below 45 years of age [13].

Furthermore, few studies have simultaneously examined individual, behavioural, reproductive, socioeconomic, and contextual determinants to explain age-related differences in HPV infection patterns. This evidence gap limits the ability of national cervical cancer prevention programs to design age-specific and life-course-appropriate interventions. Therefore, this study aimed to assess the differential patterns and determinants of HPV infection between women aged ≤40 years and those aged ≥41 years in Western Province, Zambia

## Materials and methods

### Ethics statement

The ethical clearance was obtained from the University of Lusaka on the 15^th^ of October 2025 (Ref Number: FWA00033228–03911/25). To ensure privacy and confidentiality, the data were de-identified. Permission to conduct the study was granted by the Ministry of Health, Western Provincial Health Office. Written informed consent was obtained from all participants before enrollment. The study was conducted in accordance with the principles of the Declaration of Helsinki (2013) and relevant national ethical guidelines. All data were anonymized at the point of collection, and no information capable of identifying individual participants was recorded or retained. The study was designed and reported in accordance with the Strengthening the Reporting of Observational Studies in Epidemiology (STROBE) guidelines for cross-sectional studies supplementary file (S1 Checklist).

### Study design and setting

Between 31^st^October 2025 and 20^th^January 2026, a cross-sectional study was conducted in Western Province, which comprises 16 administrative districts and has an estimated population of approximately 1,375,604 residents. The provincial health system includes over 380 health facilities, three general hospitals, and 16 laboratories providing primary healthcare services. Cervical cancer screening is available across all health facilities in the province.

### Study population

Women attending cervical cancer screening clinics in Western Province were recruited between 31^st^ October 2025 and 20^th^ January 2026. The province is home to individuals from diverse ethnic backgrounds. Recruitment was conducted in accordance with strict ethical standards, ensuring that participation was completely voluntary and free from any form of coercion. Individuals who chose not to participate were attended to through normal routine screening without, disadvantaging them because of their decision not to take part in the study

### Eligibility criteria

Eligible participants were 15 years or older, had no history of hysterectomy, were sexually active via vaginal intercourse and were willing to provide written informed consent and assent (with guardian consent for minors). Exclusion criteria included females younger than 15 years or older, but who had a hysterectomy, pregnant women, and individuals who were critically ill at the time of data collection.

### Sample size

The sample size was determined using Yamane’s (1969) formula (for a finite population). n = N/(1 + N(e)^2^ where N represents the population size. Assuming a conservative prevalence of 50% (to maximize the required sample size) and a margin of error of ±3.5%, the minimum sample size required for the study was determined to be 1,132 participants.

Participants were recruited using consecutive sampling, whereby all eligible women attending cervical cancer screening clinics during the study period were approached sequentially until the target sample size was achieved. Due to the non-probability nature of consecutive sampling, the findings are primarily generalizable to women attending the selected screening facilities

### Sample collection, storage and transportation

#### Sample collection.

HPV samples were collected using provider-collected or self-collected methods. For provider collection, a trained healthcare provider inserted a sterile speculum to visualize the cervix and rotated an HPV cervical brush 2–3 times in the cervical canal to obtain epithelial cells from the transformation zone. The brush was then placed into an Aptima collection kit and stored at 2–30 °C, remaining stable for up to 30 days before processing. For self-collection, participants received a sterile HPV kit and were instructed on proper technique, including hand hygiene, avoiding sampling during menstruation, and adopting a comfortable standing or squatting position while rotating the brush 2–3 times to collect cells. After collection, all samples were carefully placed in labeled vials in accordance with ethical and hospital standards. Both methods ensured adequate cell sampling, minimized contamination, and preserved DNA integrity, providing reliable material for HPV genotyping.

### HPV sample processing and high-risk genotyping

A 500 µL aliquot of each exfoliated cervical sample was processed on the Hologic Panther platform (Hologic, San Diego, USA), a fully automated system that performs cell lysis, removes PCR inhibitors, and binds HPV DNA to magnetic particles, which are washed before elution for PCR amplification. High-risk HPV (HR-HPV) genotyping was performed using Transcription-Mediated Amplification (TMA), detecting 14 high-risk genotypes (16, 18, 31, 33, 35, 39, 45, 51, 52, 56, 58, 59, 66, 68). Amplified products were detected using the Hybridization Protection Assay (HPA) with chemiluminescent-labelled probes. The assay incorporates an internal control to monitor target capture, amplification, and detection, while positive and negative calibrators were included in each analytical run. Runs were accepted only after meeting the manufacturer’s predefined quality control acceptance criteria; invalid runs were repeated in accordance with the manufacturer’s instructions.S1 Text and S2 Text

HPV 16 and HPV 18 were identified individually, whereas the remaining 12 high-risk genotypes were reported as a pooled result. HPV types were classified according to the WHO/IARC Monographs as definite carcinogens (Group 1: 16, 18, 31, 33, 35, 39, 45, 51, 52, 56, 58, 59), probable carcinogen (Group 2A: 68), and possible carcinogens (Group 2B: 6, 11, 26, 40, 42, 53, 54, 55, 61, 62, 64, 66, 67, 69, 70, 71, 72, 73, 81, 82, 83, 84, IS39, and CP6108) [14].

### Data collection

A structured questionnaire was used to collect sociodemographic and contextual information, including age, marital status, education level, and HPV infection status, which was categorized as either positive or negative. The questionnaire was reviewed by subject experts to ensure content validity and relevance. For participants who did not understand English, healthcare providers translated the questionnaire into Silozi to facilitate comprehension. All collected data were securely stored, with access restricted to the principal investigator and authorized research team members.

HPV knowledge was assessed using an 11-point scale based on eight multiple-choice questions containing 11 correct responses. Each correct response was assigned one point, while incorrect responses scored zero, resulting in a total score ranging from 0 to 11. Knowledge levels were categorized using Bloom’s cutoff criteria for KAP studies: scores of 80–100% were classified as high knowledge, 50–79% as medium knowledge, and below 50% as low knowledge [15].

### Data management and analysis

Data were entered into Excel and analyzed using R version 4.5.0 following data validation and cleaning. Data completeness and consistency were assessed before analysis, and variables with minimal missing observations were analyzed using complete-case analysis. Records with substantial missing information on key study variables were excluded from the final analysis. The prevalence of potential high-risk HPV infection by age group was summarized using frequencies and percentages. Factors associated with potential high-risk HPV infection were analyzed using mixed-effects logistic regression with facility-level random intercepts to account for clustering and both individual-level and contextual factors.

Variables were selected a priori based on biological relevance and existing literature. Additional variables identified in bivariate analysis (p < 0.05) were entered sequentially using a forward stepwise selection procedure to assess their contribution to model fit. To reduce limitations associated with stepwise approaches, epidemiologically important variables were retained regardless of statistical significance. Model selection and performance were evaluated using Akaike’s Information Criterion (AIC) and Bayesian Information Criterion (BIC), with lower values indicating better model fit.

A hierarchical modeling strategy was applied Model 2 included sociodemographic and behavioral factors, further adjusted for contextual and facility-level factors while accounting for clustering at the health facility/district level. Statistical significance was set at p ≤ 0.05. A 40-year age cutoff was used based on epidemiological evidence showing differences in HPV transmission dynamics, persistence, and age-specific prevalence patterns before and after mid-adulthood, including the emergence of a second peak of persistent HPV infection among older women [16,17]

## Results

### Sociodemographic characteristics of study participants

A total of 1,132 women were included in the analysis. The median number of lifetime sexual partners was 4 (IQR 3–6). Based on age stratification, 60.4%(684/1132) of women were ≤40 years of age and 39.6%(484/1132) were aged ≥ 41. Overall, 380 women 33.6%(380/1132) tested positive for HPV, while 66.4%(752/1132) tested negative. HPV infection differed significantly between the two age groups (p = 0.008), with a higher proportion of infections observed among women aged ≤40 years than among those aged ≥41 years. Vaccination coverage was low, with only 10.2% of women reporting prior vaccination, and vaccination status differed significantly by age group (p < 0.001) (Table 1).

**Table 1:** Sociodemographic characteristics of study participants by age category.

		Age-Category		
Variable (Category)	Overall n (%)	≤40 years n (%)	≥41 years n (%)	P-Value
Total	1132(100)	684(60.4)	448(39.6)	
**History Number of Partners**				
Median (IQR)	4(6-3)			
**HPV cases**				
Positive	380(33.6)	230(20.3)	150(13.3)	**0.008**
Negative	752(66.4)	454(40.1)	298(26.3)	
**Number of Children**				
≤4	797(70.4)	491(43.4)	306(27.0)	0.235
≥5	335(29.6)	193(17.0)	142(12.6)	
**Vaccination Status**				
Yes	116(10.2)	109(9.6)	7(0.6)	**<0.001**
No	1016(89.8)	575(50.8)	441(39.0)	
**Occupation**				
Employed	168(14.8)	112(9.9)	56(4.9)	
Unemployed	361(31.9)	215(19.0)	146(12.9)	0.282
Trader	205(18.1)	117(10.3)	88(7.8)	
Farmer	398(35.2)	240(21.2)	158(14.0)	
**Education level**				
Tertiary	151(13.3)	105(9.2)	46(4.1)	**<0.001**
Primary	461(40.7)	243(21.5)	218(19.2)	
Secondary	373(32.9)	246(21.7)	127(11.2)	
Uneducated	147(12.9)	90(7.9)	57(5.0)	
**Income level**				
High	47(4.1)	25(2.2)	22(1.9)	
Medium	375(33.1)	238(21.0)	137(12.1)	0.234
Low	710(62.7)	421(37.2)	289(25.5)	
**Marital Status**				
Married	450(39.8)	262(23.1)	188(16.6)	**<0.001**
Single	620(54.8)	397(35.1)	223(19.7)	
Divorced	17(1.5)	10(0.9)	7(0.6)	
Widow	45(4.0)	15(1.3)	30(2.7)	
**HPV Knowledge**				
High	324(28.6)	209(18.5)	115(10.1)	0.051
Medium	294(26.0)	184(16.3)	110(9.7)	
Low	514(45.4)	291(25.7)	223(19.7)	
**Contraceptive**				
Yes	653(57.7)	402(35.5)	251(22.2)	0.393
No	479(42.3)	282(24.9)	197(17.4)	

Data are presented as frequency. Continuous variables are summarized as median (interquartile range [IQR]). Associations between sociodemographic characteristics and age category (≤40 vs. ≥41 years) were assessed using the Chi 2 test for categorical variables. A p-value < 0.05 was considered statistically significant.

Educational attainment varied across participants, with 40.7% having primary education, 33.0% secondary education, 13.3% tertiary education, and 13.0% having no formal education. Education level differed significantly by age group (p < 0.001).

Marital status distribution showed that 39.8% of women were married, 54.8% were single, 4.0% were widowed, and 1.5% were divorced, with significant differences observed across age groups (p < 0.001). Contraceptive use was reported by 57.7% of women, while 42.3% reported no contraceptive use (Table 1).

Table 2 below shows that, in the age-stratified analysis, prevalence was higher among women aged ≤40 years (34%) than among those aged ≥41 years (33%). Among women aged ≤40 years, HPV infection was significantly associated with a higher number of children above ≥5 born by a woman, lack of HPV vaccination, occupation, lower educational attainment, low-income level, marital status, low HPV knowledge, contraceptive use, and facility location (p < 0.05).

**Table 2:** Shows the results of the chi-square test for factors associated with HPV infection, stratified by age group.

Characteristics	≤40 years(n = 684)		p-value	≥ 41 years(n = 448)		p-value
	HPV Positive n 230 (33.6%)	HPV Negative n 454(66.4%)		HPV Positive n 150 (33.4%)	HPV Negative n 298(67.5%)	
**Number of children**						
**≤4**	65 (9.5)	426 (62.3)	**<0.001**	31(6.9)	275 (61.4)	**<0.001**
**≥5**	165 (24.1)	28 (4.1)		119 (26.6)	23 (5.1)	
**Vaccination Status**						
Yes	14 (2.0)	95 (13.9)	**<0.001**	0(0)	7(1.7)	**0.136**
NO	216 (31.6)	359 (52.5)		150 (33.5)	291 (64.9)	
**Occupation**						
Employed	15 (2.2)	97(14.2)		15 (3.3)	41(9.2)	
Unemployed	73 (10.7)	142 (20.8)	**<0.001**	52 (11.6)	94 (20.9)	**<0.001**
Trader	56 (8.2)	61(8.9)		51 (11.4)	37 (8.3)	
Farmer	86 (12.6)	154(22.5)		32(7.1)	126 (28.1)	
**Education level**						
Tertiary	9 (1.3)	96 (14.0)		8 (1.8)	38 (8.5)	
Primary	110 (16.1)	133 (19.4)	**<0.001**	85 (18.9)	133 (29.7)	**<0.001**
Secondary	44 (6.4)	202 (29.5)		17 (3.8)	110 (24.5)	
Uneducated	67(9.8)	23 (3.4)		40 (8.9)	17(3.8)	
**Income level**						
High	4 (0.5)	21(3.1)		4 (0.9)	18 (4.0)	
Medium	59 (8.6)	179 (26.2)	**<0.001**	40 (8.9)	97 (21.7)	**0.092**
Low	167 (24.4)	254 (37.1)		106 (23.7)	183 (40.8)	
**Marital Status**						
Married	62 (9.1)	200 (29.2)		48 (10.7)	140 (31.2)	
Single	153 (22.4)	244 (35.7)	**<0.001**	78 (17.4)	145 (32.4)	**<0.001**
Divorced	3 (0.4)	7 (1.0)		4 (0.9)	3 (0.7)	
Widow	12 (1.8)	3 (0.4)		20 (4.5)	10 (2.2)	
**HPV Knowledge**						
High	19 (2.8)	190 (27.8)		6 (1.3)	109 (24.3)	
Medium	46 (6.7)	138 (20.2)	**<0.001**	31 (6.9)	79 (17.6)	**<0.001**
Low	165 (24.1)	126 (18.4)		113 (25.2)	110 (24.6)	
**Contraceptive**						
Yes	187 (27.3)	215 (31.4)	**<0.001**	107 (23.9)	144 (32.1)	**<0.001**
No	43 (6.2)	239 (34.9)		43 (9.5)	154 (34.3)	

Percentages were calculated using the total number of participants within each age group as the denominator (≤40 years, n = 684; ≥ 41 years, n = 448). HPV infection status was categorized as positive or negative. Associations between participant characteristics and HPV infection status were assessed using chi-square tests. P-values are reported separately for each age group. Statistical significance was defined as p < 0.05.

Among women aged ≥41 years, HPV infection was significantly associated with having more than five children, occupation, education level, marital status, HPV knowledge, contraceptive use, and facility location (all p < 0.05)

### Bivariate analysis of factors associated with hpv infection among women aged ≤ 40 years

Table 3 revealed that, in a bivariate mixed‑effects logistic regression analysis, several factors were significantly associated with HPV infection (p < 0.005).Number of children, HPV knowledge, education, occupation, Vaccination status and contraceptive.

**Table 3:** Bivariate and multivariable logistic regression analysis of factors associated with HPV Infection among women aged ≤40 years.

Variable			Model 1		Model 2	
	COR (Cl 95%)	P-Value	aOR (CI 95%)	P-Value	aOR (CI 95%)	P-value
**History Number of Partners**	1.54(1.42–1.68)	**<0.001**	1.67 (1.34–2.08)	**<0.001**	2.61 (1.27–5.86)	**<0.001**
**Number of children**						
**≤ 4**	**REF**		**REF**		**REF**	
**≥5**	37.26(23.09–60.12)	**<0.001**	6.56 (4.51– 7.14)	**<0.001**	12.44 (9.06–18.46)	**<0.001**
**Vaccination Status**						
Yes	**REF**		**REF**		**REF**	
No	4.20(2.33-7.55)	**<0.001**	0.96 (0.21–3.76)	0.887	12.23(2.69–26.53)	**0.001**
**Occupation**						
Employed	**REF**		**REF**		**REF**	
Unemployed	3.17(1.71–5.87)	**<0.001**	0.37 (0.06–2.15)	0.269	0.41 (0.07–2.39)	0.324
Trader	5.69(2.95–10.99)	**<0.001**	0.40 (0.05–3.07)	0.383	0.22 (0.02–1.86)	0.166
Farmer	3.39(1.84–6.23)	**<0.001**	0.96 (0.13– 7.18)	0.974	0.37 (0.04–3.19)	0.370
**Education level**						
Tertiary	REF		**REF**		**REF**	
Primary	8.36(4.02–17.38)	**<0.001**	1.12 (0.24–5.24)	0.879	0.61 (0.06–5.40)	0.658
Secondary	2.18(1.02–4.67)	**0.043**	0.88 (0.16–4.66)	0.889	0.87 (0.12–6.22)	0.891
Uneducated	31.55(13.58–73.28)	**<0.001**	8.84 (0.80–15.69)	0.074	4.76 (0.39–18.14)	0.221
**Income level**						
High	REF		**REF**		**REF**	
Medium	1.58(0.51–4.84)	0.420	0.61 (0.08–4.83)	0.638	0.66 (0.09–4.93)	0.693
Low	2.78(0.33–23.03)	0.343	1.79 (0.22-14.59)	0.584	3.26(1.09–9.76)	**0.034**
**Marital Status**						
Married	REF		REF		**REF**	
Single	2.39(0.92–6.19)	0.073	1.95 (0.79–4.83)	0.145	2.04(1.44–2.91)	**<0.001**
Divorced	1.35(0.34–5.39)	0.667	1.83 (0.10–33.28)	0.682	1.49 (0.08–25.22)	0.780
Widow	Omitted		Omitted		Omitted	
**HPV Knowledge**						
High	**REF**		**REF**		**REF**	
Medium	3.23(1.81–5.77)	**<0.001**	2.12 (0.55–8.15)	0.274	3.58 (0.81–15.74)	0.090
Low	12.33(7.28–20.89)	**<0.001**	7.28 (1.72–14.01)	**0.007**	1.25(0.27–5.64)	0.769
**Contraceptive**						
No	REF		**REF**		**REF**	
Yes	4.48(3.30–7.06)	<0.001	10.54 (3.70–30.82)	**<0.001**	8.38 (3.00–22.76)	**<0.001**
**Contextual Factors**						
**Visit type**						
First					**REF**	
Three years					0.39 (0.12–1.21)	0.104
**Sample collection type**						
Self					0.81 (0.31–2.11)	0.679
Provider					**REF**	
**Facility Location**						
Urban					**REF**	
Peri-Urban					0.33 (0.30–3.62)	0.365
Rural					9.23 (1.78–18.64)	**0.008**
**Districts**						
Mongu					**REF**	
Kaoma					1.50 (0.29–7.64)	0.622
Nkeyema					0.36 (0.02–6.44)	0.156
Lukulu					0.45 (0.07–2.72)	0.391
Mitete					0.10 (0.01–2.07)	0.139
Mulobezi					4.20 (0.51–34.6)	0.181
Limulunga					0.75 (0.19–2.94)	0.689
Kalabo					3.15 (0.64–15.40)	0.156
Nalolo					0.59 (0.17–1.99)	0.399
Senanga					0.61 (0.16–2.24)	0.464
Sioma					2.02 (0.30– 13.57)	0.468
Shangombo					1.94 (0.36–10.20)	0.434
Sesheke					1.21 (0.34–4.20)	0.764
Mwandi					5.27 (3.94–14.47)	**0.004**
Luampa					0.84 (0.14–5.08)	0.857
Sikongo					0.81 (0.09 –7.00)	0.857
Diagnostic Test						
AIC			289.00		283.78	
BIC			369.78		449.83	
ICC			0.528		0.251	

Women with more than five children had substantially higher odds of HPV infection compared to those with fewer than four children (cOR=37.26; 95% CI:23.09–60.12, p < 0.001). Unvaccinated women had significantly higher odds of HPV infection compared to vaccinated women (cOR=4.20% CI: 2.33–7.55, p < 0.001).

Women with primary education had higher odds of HPV infection compared with women with tertiary education (cOR = 8.36; 95% CI: 4.02–17.38). Uneducated women had elevated odds of infection (COR = 31.55; 95% CI: 13.58–73.28); however, this should be interpreted with caution. Women with secondary education also had increased odds of HPV infection (COR = 2.18; 95% CI: 1.02–4.67).Women who used hormonal contraceptives had higher odds compared to those who were not using cOR 3.69(2.78-4.88,p < 0.001).

### Multivariable analysis of factors associated with HPV infection among women aged ≤40 years

Table 3. Multivariable analysis revealed that multiple lifetime sexual partners, contraceptive use, marital status, income level and rural facility location were significantly associated with HR-HPV infection (p < 0.05) after adjusting for potential confounders and accounting for clustering effects.

Women with a higher number of lifetime sexual partners had significantly increased odds of HR-HPV infection. (aOR = 2.61; 95% CI: 1.27–5.86; p < 0.001). Similarly, women with more than five children had substantially higher odds of HR-HPV infection than those with fewer than four children (aOR = 12.44; 95% CI: 9.06–18.46; p < 0.001).

Single women had higher odds of HPV infection compared with married women (aOR = 2.04; 95% CI:1.44–2.91; p = 0.001). Furthermore, participants with low-income levels had higher odds compared to those with high income (aOR = 3.26; 95% CI: 1.09–9.76; P = 0.034)

Women reporting contraceptive use had significantly higher odds of HPV infection compared with non-users (aOR = 8.38; 95% CI: 3.00–22.76; p < 0.001). Women attending rural facilities had significantly higher odds of the HPV infection compared with those attending urban facilities (aOR = 9.23; 95% CI: 1.78–18.64; p = 0.008).

Table 3 shows geographical regional variation in high-risk HPV infection across districts in the Western Province. Significantly high infection rates were recorded in Mwandi district (aOR=5.27,95% Cl 3.94-14.47,p = 0.004) compared to Mongu District. Model fit improved progressively from Model 1 to Model 2, as evidenced by decreasing AIC and BIC values

### Bivariate analysis of factors associated with HPV infection among women aged ≥41 Years

The crude logistic regression analysis showed that several factors were significantly associated with HR-HPV infection before adjustment for potential confounders. The number of lifetime sexual partners was significantly associated with increased odds of HR-HPV infection (COR = 1.50; 95% CI: 1.36–1.66; p < 0.001). In addition, women with more than five children had higher odds of HR-HPV infection compared with those with fewer than four children (COR = 9.80; 95% CI: 5.34–17.12; p < 0.001).Furthermore, traders had significantly higher odds of HR-HPV infection compared with employed women (COR = 3.42; 95% CI: 1.63–7.19; p = 0.001). Similarly, women with primary education had higher odds of HR-HPV infection compared with those with tertiary education (COR = 2.62; 95% CI: 1.15–5.95; p = 0.021), while uneducated women had substantially higher odds (COR = 10.31; 95% CI: 3.92–27.08; p < 0.001).

Single women had higher odds of HR-HPV infection than married women (COR = 1.56; 95% CI: 1.01–2.41; p = 0.041). Widowed women also had markedly increased odds of HR-HPV infection (COR = 11.57; 95% CI: 4.11–32.56; p < 0.001), while the association among divorced women was not statistically significant. Furthermore, women with medium HPV knowledge had higher odds of infection (COR = 8.13; 95% CI: 3.02–21.88; p < 0.001), while those with low HPV knowledge had even higher odds (COR = 20.75; 95% CI: 8.14–52.88; p < 0.001). Contraceptive use was also significantly associated with HR-HPV infection, with contraceptive users having higher odds compared with non-users (COR = 2.57; 95% CI: 1.68–3.92; p < 0.001)

### Multivariable analysis of factors associated with HPV Infection among women aged ≥41 years

In the first adjusted model (Model 1), uneducated women remained significantly associated with HR-HPV infection (aOR = 4.45; 95% CI: 1.26–9.54; p = 0.003). Divorced women also had significantly increased odds of infection compared with married women (aOR = 8.76; 95% CI: 3.33–11.67; p = 0.002).

In model (Model 2), women with more than five children had significantly higher odds of HR-HPV infection compared with those with fewer than four children (aOR = 3.44; 95% CI: 2.06–8.46; p < 0.001). Unemployed women (aOR = 3.01; 95% CI: 2.01–6.90; p = 0.001) and traders (aOR = 2.45; 95% CI: 1.87–4.67; p = 0.001) were more likely to have HR-HPV infection compared with employed women. Low income was also independently associated with HR-HPV infection (aOR = 6.65; 95% CI: 3.08–15.56; p = 0.003). Women with low HPV knowledge had significantly increased odds of infection compared with women with high HPV knowledge (aOR = 12.23; 95% CI: 2.69–26.53; p = 0.001). Regarding contextual factors, women attending rural facilities had significantly higher odds of HR-HPV infection compared with those attending urban facilities (aOR = 9.23; 95% CI: 1.78–18.64; p = 0.008). In contrast, women from Nalolo district had significantly lower odds of infection compared with women from Mongu district (aOR = 0.19; 95% CI: 0.04–0.83; p = 0.028).

Model fit statistics indicated improvement in the adjusted model, with the AIC decreasing from 206.47 in the c model 1 to 182.08 in the fully adjusted model. The ICC also reduced from 0.602 to 0.469 (Table 4).

**Table 4:** Bivariate and multivariable logistic regression analysis of factors associated with HPV infection among women aged ≥ 41 years.

Variable			Model 1		Model 2	
	COR (Cl 95%)	P-Value	aOR (CI 95%)	P-Value	aOR (CI 95%)	P-value
**History Number of Partners**	1.50(1.36–1.66)	**<0.001**	1.84(0.38–2.47)	0.361	2.61 (0.34–5.86)	0.563
**Number of children**						
<4	**REF**		**REF**		**REF**	
>5	9.8(5.34–17.12)	**<0.001**	2.04(0.56–10.67)	0.563	3.44 (2.06–8.46)	**<0.001**
**Vaccination Status**						
Yes	**REF**		**REF**		**REF**	
No	Omitted		Omitted		Omitted	
**Occupation**						
Employed	**REF**		**REF**		**REF**	
Unemployed	1.34(0.67-2.67)	0.404	0.37 (0.06–2.15)	0.269	3.01(2.01–6.90)	**0.001**
Trader	3.42(1.63–7.19)	**0.001**	0.40 (0.05–3.07)	0.383	2.45(1.87–4.67)	**0.001**
Farmer	0.61(0.30–1.26)	0.190	0.96 (0.13– 7.18)	0.974	0.11(0.06–1.56)	0.115
**Education level**						
Tertiary	REF		**REF**		**REF**	
Primary	2.62(1.15–5.95)	**0.021**	5.59(0.29–7.56)	0.253	0.61 (0.06–5.40)	0.658
Secondary	0.25(0.25–1.65)	0.471	2.15(0.13–5.67)	0.582	0.87 (0.12–6.22)	0.891
Uneducated	10.31(3.92–27.08)	**<0.001**	4.45(1.26–9.54)	**0.003**	4.76 (0.39–18.14)	0.221
**Income level**						
High	REF		**REF**		**REF**	
Medium	1.65(0.52–5.28)	0.391	1.13(0.05–22.75)	0.936	2.72(0.33–22.44)	0.351
Low	2.38(0.77–7.31)	0.129	3.22(0.19–6.76)	0.415	6.65(3.08–15.56)	**0.003**
**Marital Status**						
Married	REF		REF		**REF**	
Single	1.56(1.01–2.41)	**0.041**	2.06(0.66–6.42)	0.209	2.28(0.72–7.21)	0.158
Divorced	3.85(0.83–17.87)	0.084	8.76(3.33–11.67)	**0.002**	6.96(0.61–12.56)	0.175
Widow	11.57(4.11–32.56)	**<0.001**	1.86(0.12–6.86)	0.654	2.49(0.12–13.98)	0.549
**HPV Knowledge**						
High	**REF**		**REF**		**REF**	
Medium	8.13(3.02–21.88)	**<0.001**	3.71(0.48–11.56)	0.208	3.58 (0.81–15.74)	0.090
Low	20.75(8.14–52.88)	**<0.001**	4.67(0.59–9.67)	0.143	12.23 (2.69–26.53)	**0.001**
**Contraceptive**						
No	REF		**REF**		**REF**	
Yes	2.57(1.68–3.92)	**<0.001**	1.93(0.59–6.29)	0.271	1.73(0.50–5.91)	0.381
**Contextual Factors**						
**Visit type**						
First					**REF**	
Three years					0.69 (0.20–2.33)	0.558
**Sample collection type**						
Self					1.06(0.30–3.72)	0.919
Provider					**REF**	
**Facility Location**						
Urban					**REF**	
Peri-Urban					0.48(0.01–13.05)	0.668
Rural					0.23 (0.01–4.64)	**0.708**
**Districts**						
Mongu					**REF**	
Kaoma					1.91(0.47–7.83)	0.364
Nkeyema					0.10(0.07–1.66)	0.111
Lukulu					1.21(0.17–8.24)	0.841
Mitete					Omitted	
Mulobezi					4.20 (0.51–34.6)	0.181
Limulunga					0.83(0.22–3.09)	0.790
Kalabo					0.78(0.10–6.03)	0.816
Nalolo					0.19(0.04–0.83)	**0.028**
Senanga					0.81(0.21–3.03)	0.760
Sioma					1.11(0.13–8.89)	0.920
Shangombo					0.88(0.11–6.89)	0.905
Sesheke					1.20(0.31–4.53)	0.788
Mwandi					2.51(0.41–15.11)	0.312
Luampa					1.05(0.18–6.14)	0.950
Sikongo					0.20(0.02–3.52)	0.278
Diagnostic Test						
AIC			206.47		182.08	
BIC			279.61		365.33	
ICC			0.602		0.469	

COR = Crude Odds Ratio; aOR = Adjusted Odds Ratio; CI = Confidence Interval; REF = Reference category. Bivariate and multivariable logistic regression were used to assess associations between participant characteristics and HPV infection. P-values < 0.05 were considered statistically significant. Model fit statistics: AIC = Akaike Information Criterion; BIC = Bayesian Information Criterion

## Discussion

This study provides comprehensive evidence on the age-specific patterns and determinants of HPV infection among women in Western Province, Zambia. The results offer representative epidemiological evidence for the study population and demonstrate that HPV prevalence was similar among women aged ≤40 years and those aged ≥41 years (33.6% vs. 33.4%). Overall, the study highlights the complex interplay of behavioural, socioeconomic, reproductive, and contextual factors influencing HPV epidemiology across the life course [18,19].

Previous studies have reported age-specific variations in HPV prevalence globally, with the highest prevalence typically observed among younger women, followed by a decline with increasing age [20]. In contrast, our study found comparable HPV prevalence between women aged ≤40 years and those aged ≥41 years, suggesting that HPV infection remains prevalent across both age groups in Western Province, Zambia.

The overall HPV prevalence observed in our study was comparable to that reported in Weifang, China (36.74%) and Southern Province, Zambia (35%) [6], but lower than the prevalence reported in Nigeria (82%) and South Africa (77.1%) [12,13,21], but higher than the prevalence reported in another study in China (22.03%) [22]. These differences may reflect variations in geographical location, cultural and sexual behavioural practices, study design, sample size, and the underlying burden of HIV across study populations. According to the Zambia Population-based HIV Impact Assessment (ZAMPHIA) 2021, Western Province has a substantial HIV burden, with an adult HIV prevalence of approximately 13.6%, which is higher than the national prevalence of 11.0% [23]. This elevated HIV burden may have contributed to the HPV prevalence observed in our study because HIV and HPV share common behavioural risk factors, including unprotected sexual intercourse and multiple sexual partners. In addition, a recent HIV recency study conducted in Mongu District found that 34.1% of newly diagnosed HIV infections were classified as recent [24], indicating ongoing HIV transmission within the province. HIV-induced immunosuppression has been shown to impair the clearance of HPV infections, thereby increasing the persistence of high-risk HPV genotypes and the subsequent risk of cervical precancerous lesions and cervical cancer. Collectively, these factors may have contributed to the sustained HPV burden observed across both younger and older women in Western Province.

Among women aged ≤ 40 years, several factors were significantly associated with HPV infection, including contraceptive use, multiple lifetime sexual partners, higher parity, single marital status, and rural facility location. These findings suggest that behavioural, reproductive, and contextual factors play an important role in HPV acquisition and persistence among younger women. The relatively higher HPV prevalence in this age group may be explained by greater sexual activity, increased exposure to new sexual partnerships, lower socioeconomic status, and biological factors such as cervical ectopy and hormonal changes that increase susceptibility to HPV infection. These findings are consistent with previous studies reporting that younger women are more vulnerable to HPV acquisition because of both behavioural and physiological factors [25]. The use of contraceptives and having more than five children were each associated with higher odds of HPV infection. This aligns with literature and may be attributed to repeated cervical trauma and increased hormonal changes resulting in an increased level of estrogen, even during pregnancy (20). Lack of HPV vaccination was the strongest predictor of HPV infection, with unvaccinated women demonstrating higher odds [9,26]. This supports theories of profound protectiveness and calls for 100 percent vaccination among young people. Poverty may be associated with increased vulnerability to Human Papillomavirus (HPV) infection among single young women. Economic hardship may lead some women to engage in transactional sex to sustain their livelihoods, which may increase exposure to multiple sexual partners and reduce the ability to negotiate safer sexual practices, thereby elevating the risk of HPV infection [27–29]. Heterogeneity was observed in women living in Mwandi districts, who had a significantly higher likelihood of infection, indicative of spatial clustering of HPV cases across those districts due to sexual behavior, cross-provincial mobility and other factors.

In contrast, among women aged ≥41 years, several socio-demographic and reproductive factors were significantly associated with Human Papillomavirus (HPV) infection, including parity, level of knowledge, and occupation. Women with more than five children had approximately three times higher odds of HPV infection compared with women with fewer children. This finding is consistent with previous literature and may be explained by repeated cervical trauma during childbirth, which can increase susceptibility of the cervical epithelium to HPV infection. In addition, repeated pregnancies are associated with prolonged hormonal exposure, particularly elevated estrogen levels, which may contribute to cervical epithelial changes that facilitate viral persistence [29,30].

Furthermore, the level of knowledge plays an important role in HPV prevention, as women with limited awareness about HPV transmission, vaccination, and cervical cancer screening are less likely to engage in preventive behaviors such as condom use and regular screening [31,32]. Occupation has been identified as an important socio-economic determinant of HPV infection. A study conducted in Nigeria found that unemployed women were at higher risk due to economic vulnerability, which may lead to transactional sexual relationships, limited access to HPV vaccination and cervical cancer screening, and lower health literacy [33,34]. Similarly, women engaged in informal work, such as trading, may experience irregular income and social mobility that increases exposure to multiple sexual partners, reducing opportunities for consistent preventive practices [35]. Geographical variation in HPV infection was observed in Mwandi district, suggesting possible spatial clustering among older women.

Based on the findings of this study, HPV infection in Western Province, Zambia, was associated with a combination of biological, behavioral, socio-demographic, and geographic factors that varied across age groups. Among younger women (≤40 years), HPV infection was more commonly associated with sexual behavior, contraceptive use, high parity, and lack of HPV vaccination, while among older women (≥41 years), HPV infection was more frequently associated with parity, low HPV knowledge, and occupation, particularly unemployment or informal work. Geographical variation in HPV prevalence suggested possible spatial clustering and contextual differences across districts

The study employed a relatively large and provincially representative sample of women drawn from all 16 districts of Western Province and used molecular high-risk HPV detection and genotyping. The use of mixed-effects logistic regression models accounted for clustering at facility and district levels, improving the robustness of effect estimates and allowing assessment of geographic heterogeneity.

Despite these strengths, several limitations should be acknowledged. The cross-sectional design precludes causal inference and limits the ability to establish temporal relationships between risk factors and HPV infection. Variables such as viral load and CD4 for HIV positive client were not collected

## Conclusion

This study provides comprehensive epidemiological evidence on the age-specific prevalence and determinants of HPV infection among women in Western Province, Zambia. HPV prevalence was comparable between women aged ≤40 years and those aged ≥41 years, indicating that HPV infection remains an important public health concern across the adult female population. The findings also suggest that the substantial HIV burden and ongoing HIV transmission in Western Province may contribute to the persistence of HPV infection, highlighting the importance of integrated HIV and cervical cancer prevention strategies. These results underscore the need for age-specific interventions that combine HPV vaccination, cervical cancer screening, sexual and reproductive health education, and targeted community-based prevention programmes

## Supporting information

S1 Checklist STROBE Statement—Checklist of items that should be included in reports of *cross-sectional studies.* (DOCX)

S1 Data Data 1. (XLSX)

S2 Data Data 1. (XLSX)

S1 Text Qualitative Method Verification Report Document ID:S-3. (PDF)

S2 Text SPV. (PDF)
