# Supplementary material for: Differential age-related patterns of Human Papillomavirus infection among women in Western Province, Zambia
Source: PLOS Glob Public Health. 2026 Nov 12;6(11):e0006955. doi: 10.1371/journal.pgph.0006955 (PMC13759046; doi:10.1371/journal.pgph.0006955)
Supplement: Supplementary file 1 [file pgph.0006955.s004.pdf]

Document Name: Qualitative Method Verification Report

Document ID:S-3

VERIFICATION PROTOCOL

1.0 Purpose of verification

Lewanika General Hospital Laboratory has developed a Quality Management System in line with the ISO 15189:2022. After successfully transitioning to the revised ISO 15189: 2022 standard, Lewanika General Hospital Laboratory seeks to achieve and maintain accreditation to this standard as its major objective. As part of the accreditation requirements, the laboratory is required to verify the testing methods in use to ensure that they are fit for the intended purpose.

The purpose of verifying the HPV Test using the Hologic Panther procedure was to establish that it is well documented and includes all the critical steps necessary to guide staff in detecting HPV genotypes. The verification was conducted using Hologic Panther Machine and its supplied reagents.

2.0 Principle of the Test:

The Aptima HPV 16/18/45 Genotype Assay is a fully automated nucleic acid amplification test that detects E6/E7 messenger RNA (mRNA) from high-risk HPV genotypes 16, 18, and 45. The assay consists of three sequential steps performed in a single reaction tube: target capture, Transcription-Mediated Amplification (TMA), and Hybridization Protection Assay (HPA) detection.

During target capture, HPV mRNA is released from lysed cervical cells, selectively captured by sequence-specific oligonucleotides attached to magnetic microparticles, and purified through magnetic separation and washing. The captured mRNA is then amplified using TMA, an isothermal amplification method that employs reverse transcriptase and T7 RNA polymerase to generate multiple RNA amplicons.

Amplified products are detected by HPA using chemiluminescent, genotype-specific probes, and the emitted light is measured as Relative Light Units (RLU). Final results are interpreted using signal-to-cutoff (S/CO) ratios. An internal control is included throughout the process to monitor specimen processing, nucleic acid capture, amplification, and detection, ensuring assay validity and quality control

3.0 Scope of verification:

The test under study falls under the qualitative methods and as such the study focused on verifying estimates of sensitivity and specificity of HPV Test using Hologic Panther at Lewanika General Hospital Laboratory.

The two terms are defined below:

3.1 **Sensitivity**: The probability that the test will be positive given a patient with the condition

3.2 **Specificity**: The probability that the test will be negative given a patient without a condition.

4.0 Description of the test method to be verified

The procedure for HPV testing requires Thin Prep liquid cytology specimens. The Hologic Panther uses Real-Time Polymerase Chain Reaction technology to achieve maximum sensitivity and dynamic range for detection.

5.0 Equipment to be used

5.1 Hologic Panther (V2) with Serial Number 2090002638

5.2 Rocker mixer

5.3 Bio-Safety Cabinet

5.4 Centrifuge

6.0 REAGENTS AND SUPPLIES

6.1 Aptima HPV assay kit consists of (1 assay box, 1 calibrator kit, and 1 controls kit)

6.2 Aptima Assay Fluids Kit contains Aptima Wash Solution, Aptima Buffer for Deactivation Fluid, and Aptima Oil Reagent.

6.3 Mag Wash

7.0 Specimens

20 previously tested patient samples were used in this study. The reference/expected results were provided by University Teaching Hospital an accredited laboratory. 10 of them were known negative results and 10 were known positive results. The samples were tested using a similar method at Lewanika General Hospital.

8.0 Verifier

A competent Laboratory technologist who is familiar with the procedure for HPV Test using Hologic Panther performed the Verifications.

9.0 Methodology

The study was conducted on 08/04/2025. A Laboratory Staff obtained 10 positive (HPV) and 10 negative (HPV) samples from previously tested HPV samples for 20 Patients from University Teaching Hospital Laboratory. The samples where then given to the verifier who was blinded from the results. She arranged the samples randomly. The verifier analysed the samples consecutively following the procedures for HPV Test using Hologic Panther. Results obtained were then handed over to the Verification study designer who then compiled them onto a table as shown in the results below.

The results were analyzed using the contingency tables below as follows:

**HPV Testing**	**Known Results**		**Total**
	Positive	Negative	
Positive	# true positive - TP	#false positive – FP	TP+FP
Negative	#false negative - FN	# true negative – TN	FN+TN
Total	TP+FN	FP+TN	**N**

Estimated sensitivity = 100x {TP/ (TP+FN)}

Estimated specificity = 100x {TN / (FP+TN)}

9.1 Acceptability criteria

The acceptance performance for specificity of 99.2% as per manufacturer's claim in the package insert for HPV Quant Dx assay and sensitivity 100% LGHL Laboratory set standard.

Verification Results

See attached results

10.0 Results interpretation

In this regard, according to the reference/expected results from the Verification study designer, the following table implies:

**LEWANIKA GENERAL HOSPITAL LABORATORY(ID)**	**UNIVERSITY TEACHING HOSPITAL LABORATORY(ID)**	**RESULTS**	
		**EXPECTED RESULTS (UNIVERSITY TEACHING HOSPITAL LABORATORY RESULTS)**	**VERIFICATION/ OBTAINED RESULTS (LEWANIKA GENERAL HOSPITAL LABORATORY)**
1	1	HPV Positive	HPV Positive
2	2	HPV Positive	HPV Positive
3	3	HPV Positive	HPV Positive
4	4	HPV Positive	HPV Positive
5	5	HPV Positive	HPV Positive
6	6	HPV Positive	HPV Positive
7	7	HPV Positive	HPV Positive
8	8	HPV Positive	HPV Positive
9	9	HPV Positive	HPV Positive
10	10	HPV Positive	HPV Positive
11	11	Negative HPV	Negative HPV

12	12	Negative HPV	Negative HPV
13	13	Negative HPV	Negative HPV
14	14	Negative HPV	Negative HPV
15	15	Negative HPV	Negative HPV
16	16	Negative HPV	Negative HPV
17	17	Negative HPV	Negative HPV
18	18	Negative HPV	Negative HPV
19	19	Negative HPV	Negative HPV
20	20	Negative HPV	Negative HPV
A total of 20 samples with unknown results were processed by two qualified laboratory technologists. The laboratory technologists were blinded to the expected results throughout the testing process to eliminate bias. No patient identifiers or personal information were collected, ensuring confidentiality and anonymity. The analysis was conducted solely for the purpose of equipment verification and validation, and no patient diagnostic or clinical decisions were based on these results.			

10.1 Calculations

10.1.1 RPR

RPR testing	**Known results**		**Total**
	**Reactive**	**Non-Reactive**	
Reactive	10 – TP	0 – FP	TP+FP = 10
Non-Reactive	0 – FN	10- TN	FN+TN = 10
**Total**	TP+FN = 10	FP+TN = 10	**N = 20**

True Non-Reactive (TN) = 10

False Non-Reactive (FN) = 0

True Reactive (TP) = 10

False Reactive (FP) = 0

Sensitivity =100x {TP/ (TP+FN)}

=100x { 10/ (10 + 0)}

= 100%

Specificity = 100x {TN/ (TN+FP)}

=100x {10/ (10 + 0)}

= 100%

Results acceptability: The sensitivity for the laboratory obtained results was 100% and the specificity was 100%. Therefore, the results obtained met the manufacturer's claim for Specificity of above 99.2% as documented in the Aptima HPV Quant Dx assay package insert and laboratory set 100% sensitivity standard.

11.0 Comparison of Self-Collected Vaginal and Provider-Collected Cervical HPV Test Results

Self-collected vaginal specimen	Provider-collected cervical specimen	RESULTS	
		EXPECTED RESULTS	VERIFICATION/ OBTAINED RESULTS
1	1	HPV Positive	HPV Positive
2	2	HPV Positive	HPV Positive
3	3	HPV Positive	HPV Positive

4	4	HPV Positive	HPV Positive
5	5	HPV Positive	HPV Positive
6	6	HPV Negative	HPV Negative
7	7	HPV Negative	HPV Negative
8	8	HPV Negative	HPV Negative
9	9	HPV Negative	HPV Negative
10	10	HPV Negative	HPV Negative
Validation of the self-collection method: The compatibility of the Viba cytobrush, Aptima liquid transport medium, and vaginal self-collection procedure with the Hologic Aptima HPV assay used in this study was validated before implementation. The validation addressed the suitability of the collection device and specimen medium for testing vaginally self-collected specimens			

11.1Calculations

11.2RPR

RPR testing	**Known results**		**Total**
	**Reactive**	**Non-Reactive**	
Reactive	10 – TP	0 – FP	TP+FP = 10
Non-Reactive	0 – FN	10- TN	FN+TN = 10
**Total**	TP+FN = 10	FP+TN = 10	**N = 20**

True Non-Reactive (TN) = 5

False Non-Reactive (FN) = 0

True Reactive (TP) = 5

False Reactive (FP) = 0

Sensitivity =100x {TP/ (TP+FN)}

=100x { 5/ (5 + 0)}

= 100%

Specificity = 100x {TN/ (TN+FP)}

=100x {5/ (5 + 0)}

= 100%

12 Conclusion

Test results were acceptable under the acceptance criteria set for sensitivity and specificity. It can be concluded that the procedure is well documented to guide staff to reproduce expected results. Therefore, LGHL management approves the use of the procedures for analysing HPV samples as they are fit for intended use.

13. REFERENCES

13.1 ISO15189:2022 Medical laboratories-particular requirements for quality and competence

13.2 Procedure for Method Verification (MP-06)
