# Supplementary material for: Differential age-related patterns of Human Papillomavirus infection among women in Western Province, Zambia
Source: PLOS Glob Public Health. 2026 Nov 12;6(11):e0006955. doi: 10.1371/journal.pgph.0006955 (PMC13759046; doi:10.1371/journal.pgph.0006955)
Supplement: Supplementary file 2 [file pgph.0006955.s005.pdf]

Serial Number: 2090002638	System SW Version: 7.4.3.0	Date Range: 06/01/2026 00:00:00 to 06/30/2026 23:59:00

Assay: HPV

Worklist IDs:
002638-20260531-03 Version: 5.3.5.4 (For In Vitro Diagnostic Use) ML: 909847 Exp: 01/15/2027 TCR: 00538 IC Cutoff: 143558 Analyte Cutoff: 111194
002638-20260531-08 Version: 5.3.5.4 (For In Vitro Diagnostic Use) ML: 909847 Exp: 01/15/2027 TCR: 01328 IC Cutoff: 141871 Analyte Cutoff: 114207
002638-20260531-09 Version: 5.3.5.4 (For In Vitro Diagnostic Use) ML: 909847 Exp: 01/15/2027 TCR: 01449 IC Cutoff: 145656 Analyte Cutoff: 112852
002638-20260601-01 Version: 5.3.5.4 (For In Vitro Diagnostic Use) ML: 906549 Exp: 10/16/2026 TCR: 01582 IC Cutoff: 144110 Analyte Cutoff: 113692
002638-20260601-02 Version: 5.3.5.4 (For In Vitro Diagnostic Use) ML: 909847 Exp: 01/15/2027 TCR: 01324 IC Cutoff: 139717 Analyte Cutoff: 113891
002638-20260601-03 Version: 5.3.5.4 (For In Vitro Diagnostic Use) ML: 909847 Exp: 01/15/2027 TCR: 01470 IC Cutoff: 0 Analyte Cutoff: 0

Operators: Nellyt, admin

Specimen Tested: 466 | POSITIVE: 143 | Invalid: 29

Status Flag Legend:
M - Sample ID of a new test request was manually entered via the keyboard when a barcode was not readable by the instrument.
p - Assay processing error.
RDFS - Sample dispense verification failed.
sl - Data analysis error.
VVFS - LLS measurement of TCR/Sample in MTU tube falls outside the expected volume

Reviewed By: ______________ **Date:** ______ **Verified By:** ______________ **Date:** ______

HPV

Version: 5.3.5.4 (For In Vitro Diagnostic Use)
Worklist ID: 002638-20260531-03

Time	Rack-Tube #	Sample ID	IC RLU	IC Result	RLU	S/CO	Result	Flags
06/01/2026 00:57:14	298-01	Negative Calibrator - Lot 911660	283859	Valid	0	0.00	Valid	
06/01/2026 00:57:14	298-01	Negative Calibrator - Lot 911660	290104	Valid	0	0.00	Valid	
06/01/2026 00:57:14	298-01	Negative Calibrator - Lot 911660	287386	Valid	0	0.00	Valid	
06/01/2026 00:57:14	298-02	Positive Calibrator - Lot 910689	270759	Valid	1264089	11.36	Valid	
06/01/2026 00:57:14	298-02	Positive Calibrator - Lot 910689	272200	Valid	1247177	11.21	Valid	
06/01/2026 01:02:14	298-02	Positive Calibrator - Lot 910689	318390	Valid	1195218	10.74	Valid	

HPV

Version: 5.3.5.4 (For In Vitro Diagnostic Use)
Worklist ID: 002638-20260531-09

Time	Rack-Tube #	Sample ID	IC RLU	IC Result	RLU	S/CO	Result	Flags
06/01/2026 02:27:15	298-05	Negative Calibrator - Lot 911660	293234	Valid	0	0.00	Valid	
06/01/2026 02:27:15	298-05	Negative Calibrator - Lot 911660	289806	Valid	0	0.00	Valid	
06/01/2026 02:27:15	298-05	Negative Calibrator - Lot 911660	290898	Valid	0	0.00	Valid	
06/01/2026 02:27:15	298-06	Positive Calibrator - Lot 910689	279686	Valid	1270512	11.25	Valid	
06/01/2026 02:27:15	298-06	Positive Calibrator - Lot 910689	324209	Valid	1235638	10.94	Valid	
06/01/2026 02:32:14	298-06	Positive Calibrator - Lot 910689	270044	Valid	1255590	11.12	Valid	

HPV

Version: 5.3.5.4 (For In Vitro Diagnostic Use)
Worklist ID: 002638-20260531-03

Time	Rack-Tube #	Sample ID	IC RLU	IC Result	RLU	S/CO	Result	Flags
06/01/2026 06:27:15	985-04	ZMP0834230	243802	Valid	0	0.00	Negative	

HPV

Version: 5.3.5.4 (For In Vitro Diagnostic Use)
Worklist ID: 002638-20260531-08

Time	Rack-Tube #	Sample ID	IC RLU	IC Result	RLU	S/CO	Result	Flags
06/01/2026 02:17:14	298-03	Negative Calibrator - Lot 911660	283450	Valid	0	0.00	Valid	
06/01/2026 02:17:14	298-03	Negative Calibrator - Lot 911660	282690	Valid	0	0.00	Valid	
06/01/2026 02:17:14	298-03	Negative Calibrator - Lot 911660	285086	Valid	0	0.00	Valid	
06/01/2026 02:17:14	298-04	Positive Calibrator - Lot 910689	332772	Valid	1241929	10.87	Valid	
06/01/2026 02:17:14	298-04	Positive Calibrator - Lot 910689	266356	Valid	1274934	11.16	Valid	
06/01/2026 02:22:15	298-04	Positive Calibrator - Lot 910689	252098	Valid	1290045	11.29	Valid	

HPV

Version: 5.3.5.4 (For In Vitro Diagnostic Use)
Worklist ID: 002638-20260601-01

Time	Rack-Tube #	Sample ID	IC RLU	IC Result	RLU	S/CO	Result	Flags
06/01/2026 03:32:15	173-02	Negative Calibrator - Lot 906434	285971	Valid	0	0.00	Valid	
06/01/2026 03:32:15	173-02	Negative Calibrator - Lot 906434	291599	Valid	0	0.00	Valid	
06/01/2026 03:32:15	298-08	Positive Calibrator - Lot 910689	286898	Valid	1271439	11.18	Valid	
06/01/2026 03:32:15	298-08	Positive Calibrator - Lot 910689	298256	Valid	1257185	11.05	Valid	
06/01/2026 03:37:14	298-08	Positive Calibrator - Lot 910689	279507	Valid	1261139	11.09	Valid	

Time	Rack-Tube #	Sample ID	IC RLU	IC Result	RLU	S/CO	Result	Flags
06/02/2026 00:49:03	156-01	Negative Calibrator - Lot 911660	276182	Valid	0	0.00	Valid	
06/02/2026 00:49:03	156-01	Negative Calibrator - Lot 911660	281437	Valid	0	0.00	Valid	
06/02/2026 00:49:03	156-01	Negative Calibrator - Lot 911660	280686	Valid	0	0.00	Valid	
06/02/2026 00:49:03	156-02	Positive Calibrator - Lot 910689	284181	Valid	1267615	11.13	Valid	
06/02/2026 00:49:03	156-02	Positive Calibrator - Lot 910689	312978	Valid	1243449	10.91	Valid	
06/02/2026 00:54:03	156-02	Positive Calibrator - Lot 910689	241146	Valid	1285334	11.28	Valid	

Version: 5.3.5.4 (For In Vitro Diagnostic Use)
Worklist ID: 002638-20260531-09

Time	Rack-Tube #	Sample ID	IC RLU	IC Result	RLU	S/CO	Result	Flags
06/01/2026 02:27:15	298-05	Negative Calibrator - Lot 911660	293234	Valid	0	0.00	Valid	
06/01/2026 02:27:15	298-05	Negative Calibrator - Lot 911660	289806	Valid	0	0.00	Valid	
06/01/2026 02:27:15	298-05	Negative Calibrator - Lot 911660	290898	Valid	0	0.00	Valid	
06/01/2026 02:27:15	298-06	Positive Calibrator - Lot 910689	279686	Valid	1270512	11.25	Valid	
06/01/2026 02:27:15	298-06	Positive Calibrator - Lot 910689	324209	Valid	1235638	10.94	Valid	
06/01/2026 02:32:14	298-06	Positive Calibrator - Lot 910689	270044	Valid	1255590	11.12	Valid	
